# Classification of Older Adults Who Have Diabetes by Comorbid Conditions, United States, 2005–2006

**DOI:** 10.5888/pcd9.110287

**Published:** 2012-05-17

**Authors:** Neda Laiteerapong, James Iveniuk, Priya M. John, Edward O. Laumann, Elbert S. Huang

**Affiliations:** Author Affiliations: James Iveniuk, Priya M. John, Edward O. Laumann, Elbert S. Huang, University of Chicago, Chicago, Illinois.

## Abstract

**Introduction:**

Older adults who have diabetes vary widely in terms of comorbid conditions; these conditions help determine the risks and benefits of intensive glycemic control. Not all people benefit from intensive glycemic control. The objective of this study was to classify by comorbid conditions older American adults who have diabetes to identify those who are less likely to benefit from intensive glycemic control.

**Methods:**

We used latent class analysis to identify subgroups of a nationally representative sample of community-dwelling older adults (aged 57–85 y) who have diabetes (n = 750). The subgroups were classified according to 14 comorbid conditions prevalent in the older population. Using the Akaike Information Criterion, the Bayesian Information Criterion (BIC), the sample-size adjusted BIC, and the χ^2^ goodness-of-fit statistic, we assessed model fit.

**Results:**

We found 3 distinct subgroups. Class 1 (63% of the sample) had the lowest probabilities for most conditions. Class 2 (29% of the sample) had the highest probabilities of cancer, incontinence, and kidney disease. Class 3 (9% of the sample) had the highest probabilities (>90%) of congestive heart failure and myocardial infarction. Class 1 had only 0, 1, or 2 comorbid conditions, and both class 2 and class 3 had 6 or more comorbid conditions. The 5-year death rates for class 2 (17%) and class 3 (33%) were higher than the rate for class 1 (9%).

**Conclusion:**

Older adults who have diabetes, cardiovascular disease, and 6 or more comorbid conditions may represent a subgroup of older adults who are less likely to benefit from intensive glycemic control.

## Introduction

According to the American Diabetes Association (ADA), the glycemic target for most adults who have diabetes is a glycosylated hemoglobin (HbA1c) of less than 7.0% (1). Although the strategy of intensive glycemic control (defined as an HbA1c of <7%) may benefit many people who have diabetes, it may not benefit many older adults (2). Older adults who have diabetes are more heterogeneous than younger adults in terms of diabetes duration, functional ability, and comorbid conditions. The number and type of comorbid conditions may determine the risks and benefits of intensive glycemic control. The Action to Control Cardiovascular Risk in Diabetes (ACCORD) trial heightened concerns about the harms of very intensive glycemic control (defined as an HbA1c <6.5%) among older adults (3). In light of these concerns, the current challenge is to identify older adults who are likely to benefit from intensive glycemic control.

Clinical markers may help identify these subgroups of older adults. For frail adults and adults who have a life expectancy less than 5 years, the American Geriatrics Society recommends less intensive glycemic control than that recommended by ADA (2). The 2010 ADA guidelines identify comorbid conditions, diabetes duration, hypoglycemia risk, and previous failures at intensive control as considerations for less intensive glycemic control (4). Despite these recommendations, no simple approach for identifying subgroups of older adults who would benefit from intensive glycemic control has been well accepted. For health systems, the identification of subgroups is especially difficult because many relevant markers (eg, hypoglycemia risk, mortality prediction, functional status) are not readily available from electronic medical records.

Classifying older adults who have diabetes according to comorbid conditions may be a practical strategy for identifying subgroups. Unlike methods requiring additional assessments (eg, functional status), data on comorbid conditions are readily available. Comorbid conditions are also associated with life expectancy and the degree to which intensive glycemic control provides benefits (5,6). The objective of this study was to classify by comorbid conditions the population of older adults in the United States who have diabetes in order to identify older adults who are less likely to benefit from intensive glycemic control.

## Methods

### Study design

We used latent class analysis (LCA) (7,8), the categorical analog to factor analysis, to identify subgroups by comorbid condition. We also compared their clinical characteristics and 5-year death rates. Institutional review boards at the Division of the Social Sciences at the University of Chicago and National Opinion Research Center approved data collection procedures. All respondents provided informed consent before participation in the study.

### Study population

We used data from Wave 1 of the National Social Life, Health, and Aging Project (NSHAP), a longitudinal, population-based study of health and social factors of older, community-dwelling Americans (9). Wave 1, conducted between July 2005 and March 2006 in English and Spanish, consisted of 3,005 interviews of adults aged 57 to 85; it oversampled men, black and Hispanic men and women, and men and women aged 75 to 84 at the time of screening. The sampling design also took into account urbanicity, defined as the probability-proportionate-to-size (PPS) selection of US Metropolitan Statistical Areas (MSAs) and non-MSA counties. NSHAP included an in-home interview, a biomeasure collection, and a self-administered postinterview questionnaire. Details on the NSHAP survey are available elsewhere (10,11). The Wave 1 survey had a weighted response rate of 75.5%, and the postinterview questionnaire had a weighted response rate of 84% (12).

We analyzed a subsample of 750 survey participants who self-reported diagnosed diabetes or who had undiagnosed diabetes. The following question was used to determine whether participants had diagnosed diabetes: “Has a medical doctor ever told you that you have any of the following conditions: [a list of conditions includes ‘diabetes or high blood sugar’]?” Participants who had an HbA1c of 6.5% or more and who did not self-report diabetes were considered to have undiagnosed diabetes (13). HbA1c was measured via dried blood spots during the biologic sample collection; details on the blood spot collection are available elsewhere (14). We chose not to determine diabetes diagnosis by examining data on prescribed medications because diabetes medications are also used to treat glucose intolerance.

The survey also collected self-reported data on age, sex, race/ethnicity, marital status, education, and number of visits with health care professionals in the previous year. We dichotomized number of health care visits to fewer than 4 visits in the previous year and 4 or more visits. Prescribed medications, including insulin, were cataloged during the in-home interviews (15). We measured self-rated physical health by using the single-item question, “Would you say your health is excellent, very good, good, fair, or poor?” Self-rated mental health was measured by a similar question. Responses to both questions were dichotomized (excellent/very good/good vs fair/poor). During the in-home interviews, participants also self-reported whether they had difficulty with each of the following activities of daily living for more than 3 months: walking 1 block, walking across a room, dressing, bathing/showering, eating, transferring (getting in/out of bed), and toileting. We collected data on 5-year death rates for the Wave 1 sample during Wave 2 of NSHAP during 2010 and 2011.

### Latent class analysis

For LCA, we used data from all 750 survey participants. We included 14 chronic conditions that are prevalent in the older population (2,16): arthritis, cancer, congestive heart failure, dementia, depression, emphysema, falls (in the previous 12 months), hypertension, incontinence (urinary or fecal), kidney disease, myocardial infarction, obesity, stroke, and thyroid disease. All conditions were self-reported except depression and obesity, and all were assessed during the in-home interview except fall history. Depression was measured through the 11-item Iowa form of the Center for Epidemiological Studies Depression scale (CES-D). A score of 9 points or more on the CES-D was identified as depression (17). Obesity (body mass index [BMI] ≥30 kg/m^2^] was calculated from interviewer-measured height and weight (15). Fall history was assessed in the postinterview questionnaire.

We created separate variables for each unique combination of comorbid conditions among participants who had reported data for all conditions; 508 participants, 84% of the participants who returned the postinterview questionnaire, had complete data on all comorbid conditions. At most, 508 unique combinations of comorbid conditions were possible, one for each survey participant.

### Statistical analyses

We fit latent class models successively, starting with a 1-class model and then adding another class for each successive model. Using the Akaike Information Criterion (AIC), the Bayesian Information Criterion (BIC), and the sample-size adjusted BIC (ABIC), we determined the optimal number of latent classes; lower values indicated better fit. Although the BIC is the most widely used criterion for assessing LCA model fit, multiple information criteria are often used in combination to select LCA class number (18). We also determined model fit by a χ^2^ goodness-of-fit *P* value greater than .05. We estimated models using full information maximum likelihood (FIML), which computes a case-wise likelihood function over the observed data by using all available information to estimate model parameters (19). FIML provides less biased and more efficient estimates than pair-wise deletion, case-wise deletion, or similar response-pattern imputation (20). We compared model fit between 1-, 2-, 3-, and 4-class models. We used Mplus version 6 (Muthén & Muthén, Los Angeles, California) to conduct the analyses.

We used χ^2^ analysis and 1-way analysis of variance to describe class differences in sociodemographic characteristics, self-rated health, health care use, clinical characteristics, including the prevalence of multiple comorbid conditions, and 5-year death rates. We also compared classes by frequency and combinations of comorbid conditions. We used STATA version SE10.1 (StataCorp LP, College Station, Texas) to conduct these analyses. All analyses were weighted by using population weights that adjusted for the intentional oversampling of black and Hispanic participants and incorporated a nonresponse adjustment based on age and urbanicity. We adjusted standard errors for sample stratification (sampling strata independently) and clustering (sampling individuals within each of 100 primary sampling units).

## Results

### Model fit

The AIC, BIC, and ABIC decreased as the number of classes increased from 1- to 4-class models (Table 1). The AIC, BIC, and ABIC were all lowest with the 4-class model, but the AIC and ABIC only marginally decreased between the 3- and 4-class models, and the 4th class had only 7 members. The *P* value for the χ^2^ goodness-of-fit statistic was greater than .99 for all 4 models. Thus, we selected a 3-class model to distinguish between population subgroups.

**Table 1:** Model Fit Statistics for Latent Class Analysis, Study on Older Adults Who Have Diabetes, National Social Life, Health, and Aging Project, 2005–2006

No. of classes	No. of Respondents per Class				AIC	BIC	Sample-Size Adjusted BIC	*P* Value for χ^2^ Goodness-of-Fit Statistic
1	750	—	—	—	9478.5	9543.2	9498.7	>.99
2	530	220	—	—	9226.9	9360.9	9268.8	>.99
3	470	215	65	—	9182.3	9385.6	9245.9	>.99
4	469	215	59	7	9157.1	9429.7	9242.3	>.99

Abbreviations: AIC, Akaike Information Criterion; BIC, Bayesian Information Criterion.

### Class characteristics

The estimated probability of having obesity, hypertension, or arthritis was at least 45% in all 3 classes (Table 2). Most participants (n = 470, 63%) were in class 1, which was characterized by the lowest probability for nearly all conditions. The estimated probability of having dementia, congestive heart failure, emphysema, kidney disease, or stroke was less than 5% in class 1. Approximately 29% of participants (n = 215) were in class 2. Class 2 had the highest estimated probabilities of cancer, hypertension, incontinence, kidney disease, and obesity. Class 3 (n = 65, 9%) had the highest estimated probabilities of arthritis, congestive heart failure, depression, emphysema, falls, myocardial infarction, stroke, and thyroid disease. The estimated probability of congestive heart failure or myocardial infarction was more than 90% for class 3.

**Table 2:** Estimated Probabilities of Comorbid Conditions for Each Latent Class of Older Adults Who Have Diabetes, National Social Life, Health, and Aging Project, 2005–2006

Condition	Class 1 (n = 470 [63%])	Class 2 (n = 215 [29%])	Class 3 (n = 65 [9%])
Arthritis	0.45	0.74	0.83
Cancer	0.07	0.23	0.07
Congestive heart failure	0.04	0.14	1.00
Dementia	0.00	0.01	0.00
Depression	0.22	0.35	0.40
Emphysema	0.03	0.22	0.37
Fall history	0.20	0.43	0.47
Hypertension	0.61	0.91	0.82
Incontinence	0.34	0.74	0.58
Kidney disease	0.02	0.16	0.12
Myocardial infarction	0.06	0.17	0.93
Obesity	0.54	0.65	0.46
Stroke	0.03	0.21	0.28
Thyroid disease	0.07	0.22	0.30

Class 2 had the lowest average HbA1c (Class 1, 7.24%; class 2, 6.86%; class 3, 7.28%) (Table 3). A higher percentage in class 2 and class 3 reported 4 or more visits with health care professionals in the previous year than in class 1. More than half of class 2 (59%) and class 3 (78%) reported fair or poor physical health, compared with 26% of class 1. In addition, class 2 and class 3 had more difficulty than class 1 with all activities of daily living. For example, 54% of class 2 and 66% of class 3 reported difficulty walking 1 block, compared with 24% of class 1. The 5-year death rate of class 3 (33%) was almost twice the rate of class 2 (17%) and 3 times the rate of class 1 (9%).

**Table 3:** Characteristics by Latent Class of Older Adults Who Have Diabetes, National Social Life, Health, and Aging Project, 2005–2006^a^

Characteristic	Class 1	Class 2	Class 3	*P* Value
**Age, mean (SD), y**	67.3 (7.0)	69.1 (7.7)	68.7 (7.0)	.08
**Male**	268 (57)	87 (40)	38 (59)	.003
**Race/ethnicity**				
White non-Hispanic	315 (67)	160 (74)	46 (70)	.11
Black non-Hispanic	92 (19)	31 (14)	11 (16)	
Hispanic	39 (8)	22 (10)	9 (13)	
Other	24 (5)	3 (1)	1 (1)	
**Married**	310 (66)	128 (59)	35 (53)	.09
**Less than high school education**	89 (19)	52 (24)	33 (51)	<.001
**Self-reported diabetes**	286 (82)	195 (91)	62 (95)	<.001
**Insulin use**	47 (10)	42 (20)	18 (28)	<.001
**HbA1c, mean (SD)**	7.24 (0.69)	6.86 (0.60)	7.28 (0.61)	.01
**≥4 Visits to health care professional in previous year**	246 (52)	161 (75)	46 (70)	<.001
**Self-rated health**				
Fair or poor self-rated physical health	118 (26)	125 (59)	58 (78)	<.001
Fair or poor self-rated mental health	43 (9)	54 (25)	13 (17)	<.001
**Functional disability**				
Walking a block	109 (24)	118 (54)	50 (66)	<.001
Walking across room	37 (8)	58 (27)	29 (39)	<.001
Dressing	61 (13)	77 (36)	32 (43)	<.001
Bathing/showering	32 (7)	42 (19)	29 (38)	<.001
Eating	15 (3)	25 (11)	11 (15)	<.001
Transferring	35 (8)	57 (26)	18 (24)	<.001
Toileting	39 (9)	44 (20)	18 (24)	<.001
**5-year mortality**	44 (9)	38 (17)	20 (33)	<.001

Abbreviation: SD, standard deviation; HbA1c, glycosylated hemoglobin.

^a^ Values are numbers (percentages) unless otherwise specified. Columns may not sum to 100% because of rounding. *P* values were calculated based on χ^2^ tests for categorical variables and analysis of variance for continuous variables.

### Prevalence of multiple comorbid conditions

Class 2 and class 3 had more comorbid conditions than class 1 (mean number of conditions [range]: class 1, 2.6 [0–5]; class 2, 5.5 [3–9]; class 3, 6.7 [3–9]) (Figure). Only class 1 had 0, 1, or 2 comorbid conditions, and only class 2 and class 3 had 6 or more comorbid conditions.

**Figure:**
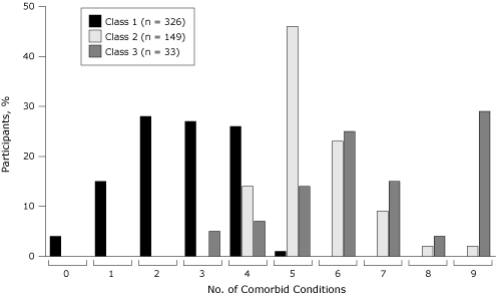
Percentage of survey participants by number of comorbid conditions. Data source: National Social Life, Health, and Aging Project, 2005–2006.

**Table:** 

No. of Comorbid Conditions	Class 1 (n = 326)	Class 2 (n = 149)	Class 3 (n = 33)
0	4	0	0
1	15	0	0
2	28	0	0
3	27	0	5
4	26	14	7
5	1	46	14
6	0	23	25
7	0	9	15
8	0	2	4
9	0	2	29

We found 266 combinations of comorbid conditions. Class 1 had 116 combinations, class 2 had 118 combinations, and class 3 had 32 combinations; we found no overlap of combinations between classes. The most frequent combinations of conditions were arthritis, hypertension, incontinence, and obesity (n = 18) and arthritis, hypertension, and obesity (n = 15).

## Discussion

In this nationally representative sample of community-dwelling older adults who have diabetes, we found 3 classes of people, based on their comorbid conditions: people in class 1 (63% of the sample) had 2 or fewer comorbid conditions and were relatively healthy; people in class 2 and class 3 were sicker; they had 6 or more comorbid conditions, a higher 5-year death rate, more health care visits, and higher rates of insulin use, functional disability, and fair or poor self-rated health. Classes were not distinguished by any single comorbid condition. However, in class 3, the estimated probability of myocardial infarction was 93% and congestive heart failure, 100%; one-third of respondents in class 3 died within 5 years of participating in the first wave of the study.

The ADA (4) recommends that clinicians use information on comorbid conditions to identify older adults who are less likely to benefit from intensive glycemic control. However, because comorbid conditions co-occurred in many different patterns across all classes, the task of identifying patients by comorbid conditions in clinical practice would be challenging. Instead, we found a potentially simpler strategy. Using 2 categories based on the number of comorbid conditions (high [≥6] and low [≤2], one could assign nearly half of the participants in our study (n = 232, 46%) to the correct class. Thus, older adults who have diabetes and 6 or more comorbid conditions would be unlikely to benefit from intensive glycemic control.

The presence of cardiovascular disease may raise concerns about the effects of intensive glycemic control. In the ACCORD trial, the presence of cardiovascular disease appeared to decrease the benefits of intensive glycemic control (3). In the Veterans Affairs Diabetes Trial, the presence of coronary calcification was associated with higher rates of cardiovascular events during very intensive glycemic control (21). These recent findings would have direct implications for our class 3 participants, for whom the estimated probability of cardiovascular disease was more than 90%. Although most participants in class 3 had cardiovascular disease, participants in class 1 and class 2 also had cardiovascular disease. Thus, decisions about intensive glycemic control should take into account both counts of comorbid disease and the presence of sentinel conditions, such as cardiovascular disease.

This research has several strengths and limitations. It demonstrates that distinct subgroups of older adults who have diabetes share patterns of comorbid conditions. It partially simplifies the complex heterogeneity of comorbid conditions among older adults who have diabetes. Our classification system, based on 2 categories, could guide future geriatric diabetes treatment recommendations. One limitation to this work is that we were unable to account for the likely important effects of diabetes duration. Differences in duration have been proposed to explain the differences between the United Kingdom Prospective Diabetes Study (22) and more recent trials. Although too late for our study, NSHAP now collects self-reported data on diabetes duration. One limitation to using self-reported data on diabetes duration, however, is the delay between diabetes onset and diabetes diagnosis; previous trials have defined the onset of diabetes by clinical diagnosis (1,3,21). Future longitudinal data would allow examination of the development of new comorbid conditions in subgroups of older adults who have diabetes. Finally, because information on fall history was collected through the postinterview questionnaire and not during the in-home interview, this information may have been systematically underreported, and the lack of these data reduced the number of people in our analysis.

Classifying the older US population who have diabetes on the basis of comorbid conditions produces clinically distinctive subgroups; however, these subgroups overlap in the predicted probability of comorbid conditions. Relying on classes based on comorbid conditions alone may not provide a definitive classification system for this population. However, using information on cardiovascular disease history and counts of comorbid conditions could provide strategies to clinicians and policy makers for distinguishing clinically important subgroups. Future research is needed to evaluate alternative strategies for identifying the subgroup of older adults who have diabetes in whom the risks of intensive glycemic control outweigh its benefits.
